# Author Correction: Evaluation of secondary sexual dimorphism of the dioecious *Amaranthus palmeri* under abiotic stress

**DOI:** 10.1038/s41598-023-41385-x

**Published:** 2023-08-29

**Authors:** Nicholas E. Korres, Jason K. Norsworthy, Toby FitzSimons, Trenton L. Roberts, Derrick M. Oosterhuis, Govindjee Govindjee

**Affiliations:** 1 School of Agriculture, Department of Agriculture, University of Ioannina, Kostakii, 47100 Arta, Greece; 2 Crop Soil and Environmental Sciences, University of Arkansas, Fayetteville, AR 72704 USA; 3 PepsiCo Inc., St. Paul, MN 55108 USA; 4 Plant Biology, Biochemistry and Biophysics, University of Illinois at Urbana-Champaign, Urbana, IL 61801 USA

Correction to: *Scientific Reports* https://doi.org/10.1038/s41598-023-40453-6, published online 12 August 2023

The original version of this Article contained an error in the Table 1 legend.

“Values with the same letter (lower case and superscript) within each column are not different at *a* = 0.05 of *A. palmeri* sex × NPK deficiency × plant organ on leaf and stem mineral content at harvest averaged across light intensities.”

now reads:

“Effects of *A. palmeri* sex × NPK deficiency × plant organ on leaf and stem mineral content at harvest averaged across light intensities. L = leaf, S = stem (incl. inflorescences). Values with the same letter (lower case and superscript) within each column are not different at *a* = 0.05.”

The original Article has been corrected.

