# Supplementary material for: Evaluation of secondary sexual dimorphism of the dioecious Amaranthus palmeri under abiotic stress
Source: Sci Rep. 2023 Aug 12;13:13156. doi: 10.1038/s41598-023-40453-6 (PMC10423251; doi:10.1038/s41598-023-40453-6)
Supplement: Supplementary file 1 — Supplementary Information. [file 41598_2023_40453_MOESM1_ESM.docx]

**Supporting Information**

**Evaluation of secondary sexual dimorphism of the dioecious *Amaranthus palmeri* under abiotic stress**

Nicholas E. Korres^1*^, Jason K. Norsworthy^2^, Toby FitzSimons^3^, Trenton L. Roberts^2^, Derrick M. Oosterhuis^2^, Govindjee Govindjee^4^.

^1^School of Agriculture, Dept. of Agriculture, University of Ioannina, Kostakii, Arta, 47100, Greece; ^2^Crop Soil and Environmental Sciences, University of Arkansas, Fayetteville, AR 72704, USA; ^3^PepsiCo Inc., St. Paul, MN 55108, USA; ^4^Biochemistry, Biophysics and Plant Biology, University of Illinois at Urbana-Champaign, Urbana, IL 61801, USA

***Email of the corresponding author:**  [nkorres@uoi.gr](mailto:nkorres@uoi.gr); [nkorres@yahoo.co.uk](mailto:nkorres@yahoo.co.uk)

**Materials and Methods**

**Plant Material**

Here we provide supplement information for Materials and Methods we used in this research. More particularly, *Amaranthus palmeri* seeds were collected from plants growing at the University of Arkansas–Agricultural Research and Extension Center, Fayetteville, AR (36.094464, -94.172074) and stored in sealed vials at 5^o^ C until planting. These seeds were planted in trays (52.5 cm × 25.5 cm × 5.5 cm) containing a commercial potting mix (Sunshine LC1, SunGro Horticulture, Agawam, MA) and grown in a greenhouse with a 35^o^/23 ^o^C day/night temperature and a 14-h/10-h light/dark period. The intensity of the ambient white light ranged from 1000 to 1500 μmol photons m^−2^ s^−1^. One hundred vigorous *A. palmeri* seedlings at one-two leaf growth stage, without any sign of injury or damage transplanted into Jiffy pots (Jiffy-7® peat pellets, Hummert TM International, Earth City, MO), and placed in 72-plug plastic trays until the 6- to 8-leaf growth stage. Each plant was then moved to a polyvinylchloride pot (37-cm height by 30-cm diameter) containing a commercial potting mixture and grown under greenhouse conditions similar to those described above, until the reproductive stage.

Water-soluble all-purpose plant food (Scotts Miracle-Gro Products, Marysville, OH) containing 24%, N 8% P, and 16% K was provided every 10 to 14 d. When the sex of *A. palmeri* plants could be distinguished, cuttings were obtained from 50 different parental plants of each sex and allowed to root and grow for 10 d in a growth chamber (CMP 6050, Conviron, Winnipeg, Canada). After this period, young seedlings of similar size were moved into individual 37 by 30 cm pots containing a 3:1:0.5:0.5 substrate mixture of washed sand (Oldcastle® Lawn & Garden, Hope, AR), vermiculite, perlite, and Sunshine® Canadian sphagnum peat moss (SunGro Horticulture, Agawam, MA) and placed into three Conviron growth chambers, each of which provided a different intensity of white light, under a 14-h/10-h light / dark period and 35^o^/25 ^o^C day/night temperature.

**Experimental Design and Treatments**

A factorial experiment (i.e., 3 levels of white light intensity × 3levels of mineral deficiency × 2 *A. palmeri* sex) arranged as a randomized complete block design was conducted, using different intensities of white light (in growth chamber, the blocking treatment), for both male and female *A. palmeri* plants, with N, or P or K deficiency (henceforth NPK deficiency) as randomized treatments within each block. Ten plants were used for each combination of light intensity, NPK deficiency and *A. palmeri* sex. We used 3 different light intensities: Low 150 μmol photons m^−2^ s^−1^ (low); 450 μmol photons m^−2^ s^−1^ (medium) and 1300 μmol photons m^−2^ s^−1^ (high). Sage and Pearcy^1^ observed similar growth characteristics of plants, including redroot pigweed (*Amaranthus retroflexus* L.) and common lambsquarters (*Chenopodium album* L.), at growth room light intensity of 600 μmol photons m^−2^ s^−1^, although well below the maximum light intensity that occurs in the field, with these grown in the field.

Each NPK deficiency treatment had, as it is mentioned in the main body text of this article, only 10% N, P, or K of the standard N (91.4 g NH_4_NO_3_ L^−1^), P (40.3 g NaH_2_PO_4_ ×2H_2_O L^-1^) or K (71.4 g K_2_SO^4^ L^−1^) stock solutions, as described in Yoshida et al.^2^, and as recommended by the N-STaR Lab at the University of Arkansas, Altheimer Lab, Fayetteville, AR (T.L. Roberts, personal communication). The above solution was modified to contain all other essential minerals, but only 10% N or 10% P or 10% K of the standard N, P, and K stock solution (as stated above). We note that Das and Sen^3^ had used a similar approach to induce mineral deficiency in *Cicer arietinum* L. (chickpea). Hence, for each light-intensity used, each of the 20 *A. palmeri cuttings* (10 cuttings from male parents and 10 cuttings from female parents) were supplied with 300 ml of a N, K, or P deficient solution, every 2 d. Plants were daily rotated within each growth chamber to avoid possible shading effects. Also, 10 plants from each *A. palmeri* sex receiving full nutrition, were left to grow under greenhouse conditions (i.e., 12- to 14-h/12- to 10-h light/dark period with 32^o^/25 ^o^C day/night temperature and ambient light intensity, which ranged from 1000 to 1500 μmol photons m^−2^ s^−1^, and used as controls. The above set of experimental treatments was conducted twice.

**References**

1. Sage, R. F. & Pearcy, R. W. The nitrogen use efficiency of C3 and C4 plants. II. Leaf nitrogen effects on the gas exchange characteristics of *Chenopodium album* (L.) and *Amaranthus retroflexus* (L.). *Plant Phys*. 84, 959-963 (1987)
2. Yoshida, S., Forno, D. A., Cock, J. H. & Gomez K. A. Laboratory Manual for Physiological Studies of Rice. 3rd ed. (International Rice Research Institute, 1976) pp. 61–65.
3. Das, B. K. & Sen P. Effect of nitrogen, phosphorus and potassium deficiency on the uptake and mobilization of ions in Bengal gram (*Cicer arietinum*). *J. Biosci.* 3, 249-258 (1981).
4. Kautsky, H. and Hirsch, A. (1931) Naturwissenschaften 19, 964

**
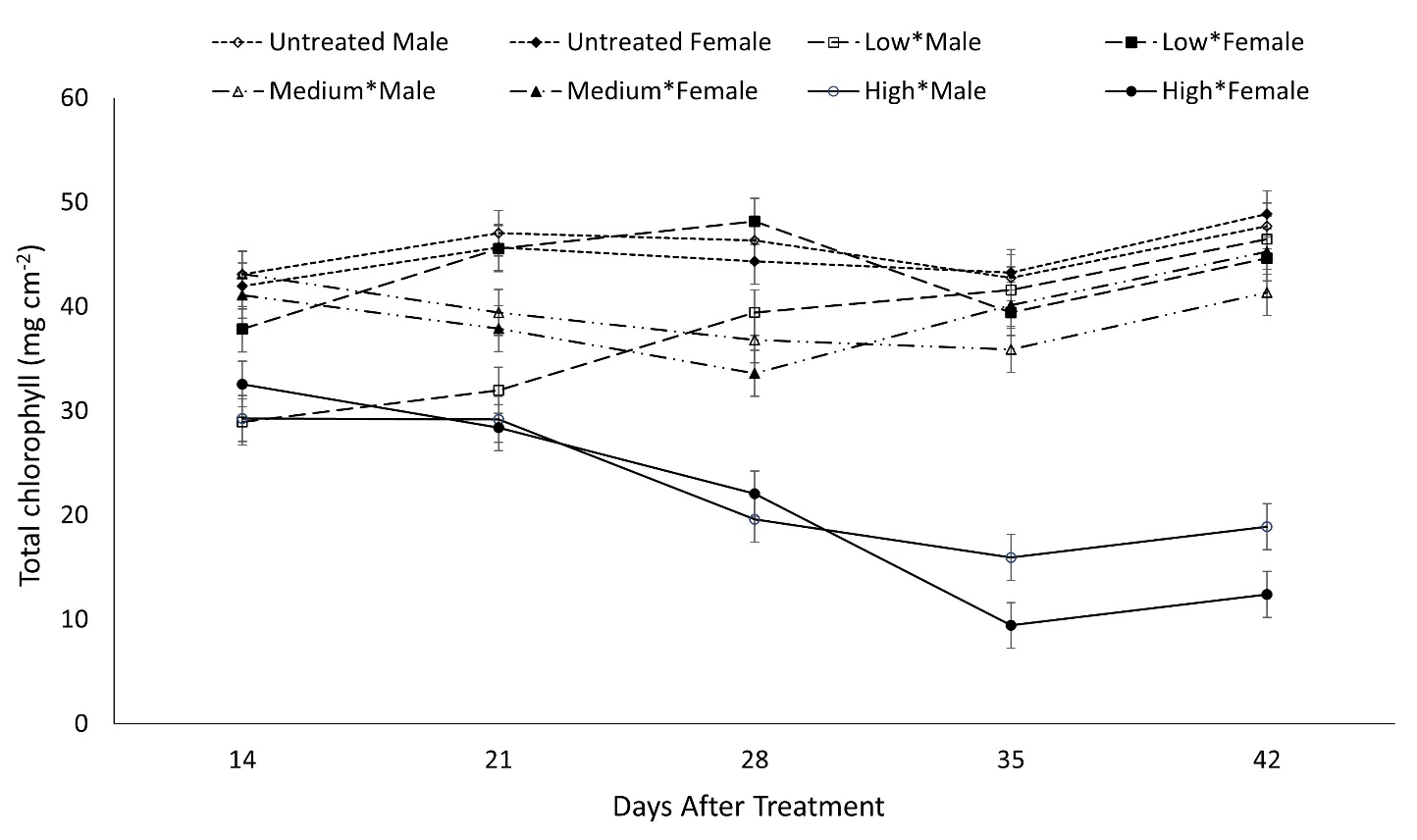
**

**Figure S1.** Leaf total chlorophyll in male and female *A. palmeri* plants of untreated plants and at 3 different white light intensities as measured throughout the experimental period (DAT) and averaged across NPK deficiency treatments. Untreated controls were grown at ambient light whereas low, medium, and high refer to 150, 450 and 1300 µmol photons m^-2^ s^-1^ (of light) respectively for male or female plants. Untreated controls are depicted with rhombus (open for males and closed symbol for females) (see dotted line). Squares represent the total chlorophyll content in male plants (open squares), and in female plants (closed squares) both under low light intensity (150 μmol photons m^-2^ s^-1^) (see dashed line). Open triangles are for the data from the male plants, and closed triangles are for female plants, both under medium light intensity (450 μmol photons m^-2^ s^-1^) (long dashed line with dots). The total chlorophyll content under high light intensity (1300 μmol photons m^-2^ s^-1^) (solid line) is shown for both the male (open circles) and the female (closed circles) plants. Vertical bars represent LSD=Least Significant Differences at *a*=0.05.

**
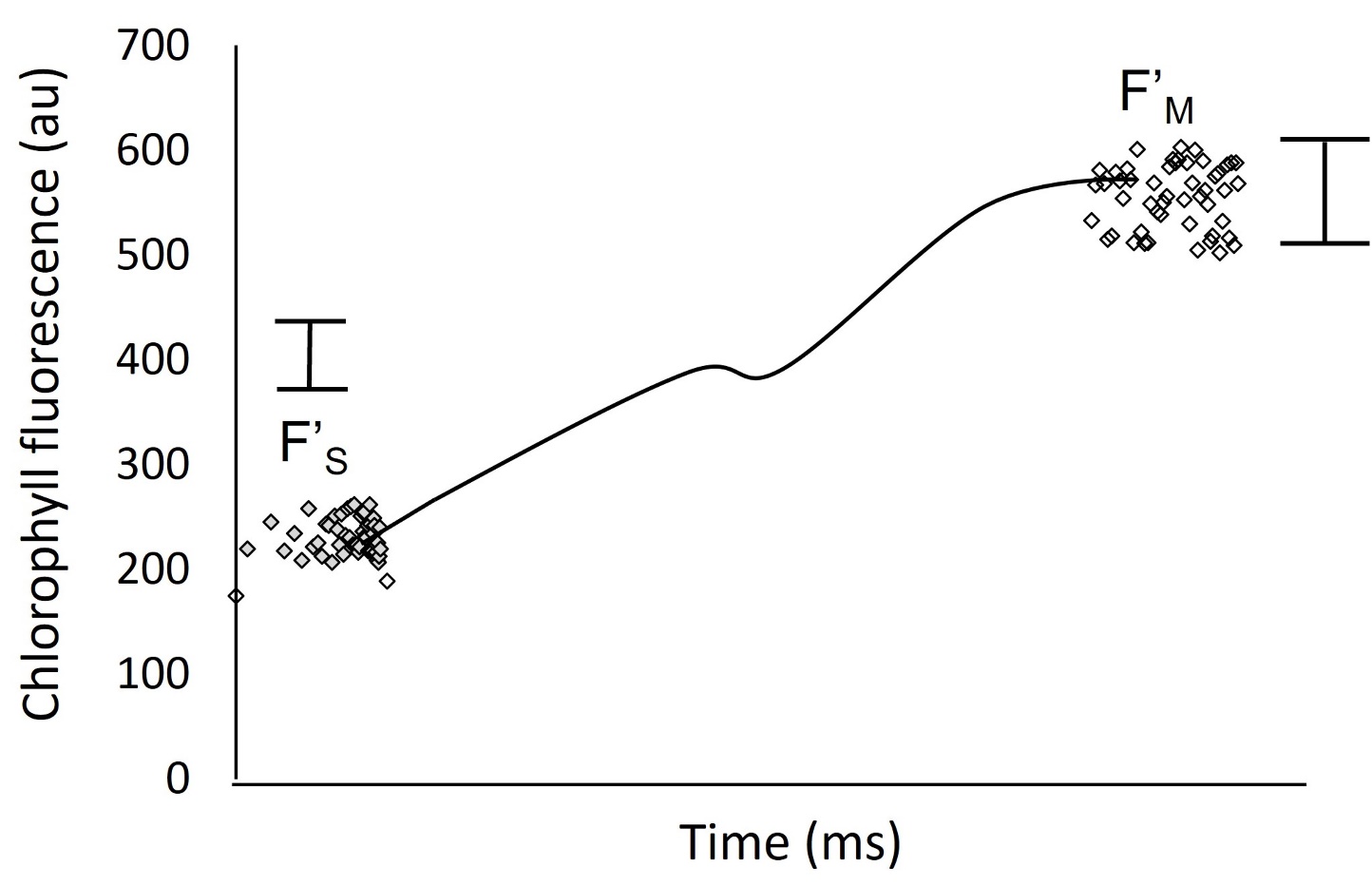
**

**Figure S2.** Chlorophyll fluorescence measurements with a “hypothetical manually” fitted curve (Kautsky and Hirsch)^4^ in light adapted leaves of *Amaranthus palmeri* grown under greenhouse conditions with ambient light and no NPK deficiency (untreated controls). Each measurement represents an individual *Amaranthus palmeri* plant (male or female) throughout the experimental period (see M&M). Vertical bars represent the Least Significance Difference (LSD) for F’_S_ and F’_M_ at *a*=0.05; au=arbitrary units, ms=milliseconds

**
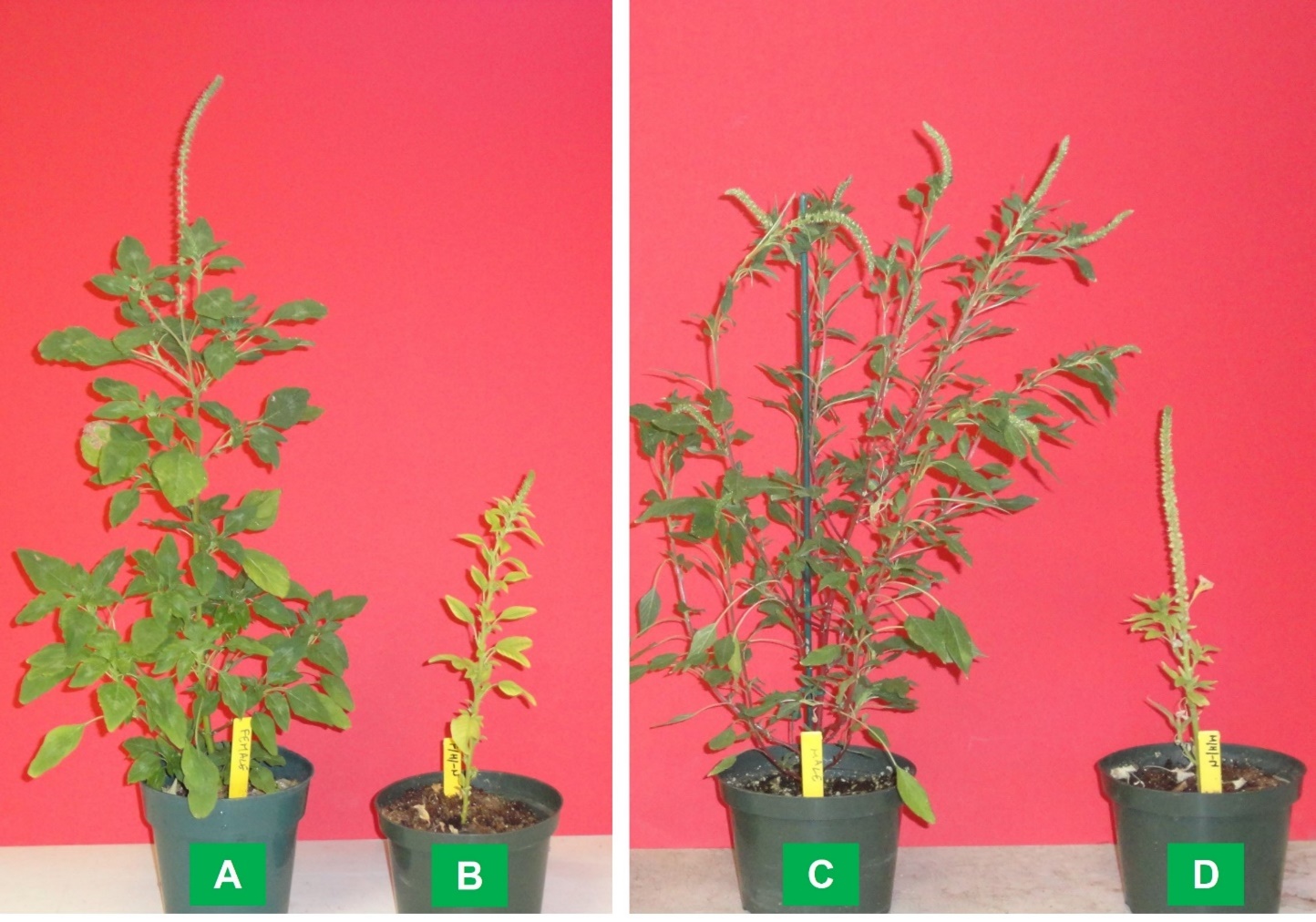
**

**Figure S3**. (A) Female and (C) male *A. palmeri* plants grown under greenhouse conditions with ambient light and no NPK deficiency (untreated controls) compared with (B) a female and (D) a male *A. palmeri* plant grown under high white-light intensity (1300 μmol photons m^-2^ s^-1^) and N deficiency.

**Table S1.** ANOVA table depicting the effects of light intensity, NPK deficiency, *Amaranthus palmeri* sex, plant organ and their interactions on organ (leaf vs. stem + inflorescence) mineral content at harvest

| Variable | L×NPK | L×G | NPK×G | L×PO | NPK×PO | G×PO | L×NPK×G | L×NPK×PO | NPK×G×PO | L×G×PO | L×NPK×G×PO |
| --- | --- | --- | --- | --- | --- | --- | --- | --- | --- | --- | --- |
| N | 0.938^ns^ | 0.133^ns^ | 0.003** | 0.600^ns^ | <.0001 | <.0001 | 0.129^ns^ | 0.885^ns^ | 0.007** | 0.499^ns^ | 0.533^ns^ |
| P | 0.026* | 0.782^ns^ | 0.012* | 0.581^ns^ | <.0001 | <.0001 | 0.134^ns^ | 0.454^ns^ | 0.023* | 0.943^ns^ | 0.533^ns^ |
| K | 0.133^ns^ | 0.984^ns^ | 0.001** | 0.842^ns^ | <.0001 | <.0001 | 0.869^ns^ | 0.807^ns^ | 0.009* | 0.976^ns^ | 0.795^ns^ |
| Ca | <.0001 | 0.947^ns^ | 0.0025* | <.0001 | <.0001 | <.0001 | 0.757^ns^ | 0.005** | 0.0038** | 0.737^ns^ | 0.789^ns^ |
| Mg | 0.0037** | 0.805^ns^ | 0.0456* | 0.0005*** | <.0001 | <.0001 | 0.629^ns^ | 0.087^ns^ | 0.0209* | 0.798^ns^ | 0.692^ns^ |
| S | 0.001** | 0.829^ns^ | 0.005* | 0.003* | <.0001 | <.0001 | 0.789^ns^ | 0.030* | 0.011* | 0.794^ns^ | 0.945^ns^ |
| Na | 0.224^ns^ | 0.102^ns^ | 0.002* | 0.724^ns^ | <.0001 | 0.0002*** | 0.265^ns^ | 0.708^ns^ | 0.017* | 0.467^ns^ | 0.674^ns^ |
| Fe | 0.151^ns^ | 0.102^ns^ | 0.599^ns^ | 0.479^ns^ | 0.041* | 0.227^ns^ | 0.433^ns^ | 0.713^ns^ | 0.705^ns^ | 0.528^ns^ | 0.947^ns^ |
| Mn | 0.047* | 0.056^ns^ | 0.013* | 0.010** | <.0001 | <.0001 | 0.212^ns^ | 0.166^ns^ | 0.029* | 0.177^ns^ | 0.473^ns^ |
| Zn | 0.0012** | 0.101^ns^ | 0.012* | 0.0009*** | <.0001 | <.0001 | 0.073^ns^ | 0.021* | 0.036* | 0.221^ns^ | 0.146^ns^ |
| Cu | 0.672^ns^ | 0.643^ns^ | 0.087^ns^ | 0.652^ns^ | 0.0103* | 0.191^ns^ | 0.410^ns^ | 0.812^ns^ | 0.231^ns^ | 0.791^ns^ | 0.597^ns^ |
| B | 0.277^ns^ | 0.879^ns^ | 0.189^ns^ | 0.078^ns^ | <.0001 | <.0001 | 0.175^ns^ | 0.442^ns^ | 0.095^ns^ | 0.988^ns^ | 0.516^ns^ |

L=light intensity, NPK=NPK deficiency, G=*A. palmeri* sex and PO=plant organ (stem + inflorescence, leaf). *=significant at *a*=0.05; **=significant at *a*=0.01; ***significant at *a*=0.001; <.0001=significant at *a*=0.0001; ns=no significant.

**Table S2.** Partial correlation matrix of leaf mineral content of *Amaranthus palmeri* female and male plants. Numbers highlighted in red and blue are positively and negatively correlated respectively at *a*=0.1 probability level.

|  | N | P | Kt | Ca | Mg | S | Na | Fe | Mn | Zn | Cu | B |
| --- | --- | --- | --- | --- | --- | --- | --- | --- | --- | --- | --- | --- |
| *A. palmeri* female plants | | | | | | | | | | | | |
| N | . | 0.143 | 0.119 | 0.351 | 0.053 | -0.428 | -0.196 | 0.052 | 0.021 | -0.314 | 0.556 | 0.225 |
| P |  |  | -0.640 | -0.007 | -0.026 | -0.138 | 0.510 | -0.392 | 0.255 | -0.381 | 0.398 | 0.358 |
| K |  |  | . | -0.304 | 0.250 | -0.041 | 0.549 | -0.268 | 0.161 | -0.065 | 0.330 | 0.307 |
| Ca |  |  |  |  | 0.576 | 0.360 | 0.259 | -0.298 | 0.286 | 0.361 | -0.232 | -0.102 |
| Mg |  |  |  |  | . | 0.051 | -0.136 | -0.004 | 0.084 | -0.203 | 0.058 | 0.405 |
| S |  |  |  |  |  |  | -0.062 | -0.323 | 0.405 | -0.595 | 0.680 | 0.313 |
| Na |  |  |  |  |  |  | . | 0.387 | -0.183 | -0.003 | -0.037 | 0.074 |
| Fe |  |  |  |  |  |  |  |  | 0.872 | -0.483 | 0.423 | 0.116 |
| Mn |  |  |  |  |  |  |  |  | . | 0.644 | -0.524 | -0.201 |
| Zn |  |  |  |  |  |  |  |  |  | . | 0.778 | 0.169 |
| Cu |  |  |  |  |  |  |  |  |  |  | . | -0.418 |
| B |  |  |  |  |  |  |  |  |  |  |  | . |
| *A. palmeri* male plants | | | | | | | | | | | | |
| N | . | 0.732 | 0.838 | 0.396 | -0.402 | -0.088 | 0.069 | 0.337 | -0.277 | -0.375 | 0.660 | 0.393 |
| P |  |  | -0.548 | -0.281 | 0.708 | -0.172 | 0.092 | -0.275 | 0.389 | -0.070 | -0.569 | -0.133 |
| K |  |  | . | -0.646 | 0.331 | 0.491 | 0.091 | -0.542 | 0.502 | 0.362 | -0.311 | -0.564 |
| Ca |  |  |  |  | 0.498 | 0.737 | 0.054 | -0.215 | 0.284 | 0.399 | -0.027 | -0.291 |
| Mg |  |  |  |  | . | -0.020 | -0.069 | 0.052 | -0.312 | 0.174 | 0.290 | 0.284 |
| S |  |  |  |  |  |  | 0.132 | 0.175 | -0.299 | -0.136 | -0.431 | 0.458 |
| Na |  |  |  |  |  |  | . | 0.182 | -0.048 | -0.051 | 0.050 | -0.004 |
| Fe |  |  |  |  |  |  |  |  | 0.704 | 0.159 | -0.165 | -0.423 |
| Mn |  |  |  |  |  |  |  |  | . | 0.302 | 0.118 | 0.597 |
| Zn |  |  |  |  |  |  |  |  |  |  | 0.390 | -0.109 |
| Cu |  |  |  |  |  |  |  |  |  |  | . | -0.015 |
| B |  |  |  |  |  |  |  |  |  |  |  | . |

**Table S3.** Partial correlation matrix of stem mineral content of *Amaranthus palmeri* female and male plants. Numbers highlighted in red and blue are positively and negatively correlated respectively at *a*=0.1 probability level.

|  | N | P | K | Ca | Mg | S | Na | Fe | Mn | Zn | Cu | B |
| --- | --- | --- | --- | --- | --- | --- | --- | --- | --- | --- | --- | --- |
| *A. palmeri* female plants | | | | | | | | | | | | |
| N | . | 0.181 | 0.053 | 0.408 | 0.225 | -0.384 | -0.030 | 0.421 | -0.242 | -0.274 | 0.491 | 0.088 |
| P |  |  | -0.382 | -0.026 | 0.106 | -0.145 | 0.310 | -0.302 | 0.249 | -0.462 | 0.463 | 0.231 |
| K |  |  | . | -0.012 | 0.450 | -0.238 | 0.410 | 0.103 | -0.025 | -0.271 | 0.390 | -0.048 |
| Ca |  |  |  |  | 0.181 | 0.542 | -0.002 | -0.441 | 0.379 | 0.554 | -0.382 | 0.198 |
| Mg |  |  |  |  | . | 0.245 | -0.292 | -0.253 | 0.287 | 0.087 | -0.115 | 0.490 |
| S |  |  |  |  |  |  | 0.125 | 0.140 | 0.108 | -0.527 | 0.564 | 0.028 |
| Na |  |  |  |  |  |  | . | 0.072 | -0.005 | 0.211 | -0.156 | 0.258 |
| Fe |  |  |  |  |  |  |  |  | 0.712 | 0.061 | -0.013 | 0.215 |
| Mn |  |  |  |  |  |  |  |  | . | 0.254 | -0.255 | -0.343 |
| Zn |  |  |  |  |  |  |  |  |  |  | 0.779 | -0.160 |
| Cu |  |  |  |  |  |  |  |  |  |  | . | -0.160 |
| B |  |  |  |  |  |  |  |  |  |  |  | . |
| *A. palmeri* male plants | | | | | | | | | | | | |
| N | . | 0.506 | 0.847 | 0.120 | 0.005 | -0.188 | 0.439 | 0.239 | -0.178 | -0.180 | 0.424 | 0.500 |
| P |  |  | -0.376 | -0.130 | 0.558 | -0.150 | -0.197 | -0.126 | 0.178 | -0.224 | -0.249 | -0.017 |
| K |  |  | . | -0.346 | -0.030 | 0.530 | -0.187 | -0.459 | 0.445 | 0.144 | -0.098 | -0.653 |
| Ca |  |  |  |  | 0.455 | 0.712 | 0.085 | -0.081 | 0.121 | 0.403 | 0.081 | -0.092 |
| Mg |  |  |  |  | . | 0.073 | 0.070 | -0.078 | -0.087 | 0.118 | 0.083 | 0.022 |
| S |  |  |  |  |  |  | 0.009 | 0.206 | -0.353 | -0.095 | -0.394 | 0.452 |
| Na |  |  |  |  |  |  | . | 0.116 | 0.069 | -0.127 | -0.174 | -0.204 |
| Fe |  |  |  |  |  |  |  |  | 0.692 | 0.053 | -0.018 | -0.484 |
| Mn |  |  |  |  |  |  |  |  | . | 0.388 | -0.099 | 0.675 |
| Zn |  |  |  |  |  |  |  |  |  |  | 0.463 | -0.191 |
| Cu |  |  |  |  |  |  |  |  |  |  | . | 0.044 |
| B |  |  |  |  |  |  |  |  |  |  |  | . |
|  |  |  |  |  |  |  |  |  |  |  |  |  |

**Table S4.** ANOVA table depicting the effects of light intensity, NPK deficiency, *Amaranthus palmeri* sex and their interactions on chlorophyll *a*, chlorophyll b and chl *a*/b ratio during the experimental period

| Response variable | T | G | T $\times$ G | L $\times$ G | NPK $\times$ G | T $\times$ L $\times$ G | T $\times$ NPK $\times$ G | L $\times$ NPK $\times$ G |
| --- | --- | --- | --- | --- | --- | --- | --- | --- |
| Chlorophyll *a* | <0.0001 | 0.0007^**^ | 0.268^ns^ | 0.018^*^ | 0.362^ns^ | <0.0001^***^ | 0.563^ns^ | 0.0007^**^ |
| Chlorophyll b | <0.0001 | 0.0006^**^ | 0.306^ns^ | 0.016^*^ | 0.321^ns^ | <0.0001^***^ | 0.545^ns^ | 0.0006^**^ |
| Chlorophyll a/b | <0.0001 | 0.836^ns^ | 0.056^ns^ | 0.309^ns^ | 0.502^ns^ | 0.002** | 0.962^ns^ | 0.532^ns^ |

T=sampling time; G=*A. palmeri* sex; L=light intensity; NPK=NPK deficiency; *=significance level *a*=0.05; **=significance level *a*=0.01; ***=significance level *a*=0.001; ns=no significant.

**Table S5.** ANOVA table depicting the effects of *Amaranthus palmeri* sex, light intensity (including untreated plants), sampling time and their interactions on total chlorophyll content.

| Response variable^†^ | T | G | L | T×G | T×L | G×L | T×G×L |
| --- | --- | --- | --- | --- | --- | --- | --- |
| Total chlorophyll | <0.0001^***^ | 0.216^ns^ | <0.0001^***^ | 0.0468^*^ | <0.0001^***^ | 0.133^ns^ | <0.0001^***^ |

^†^T=sampling time; G=*A. palmeri* sex; L=light intensity; *=significance level *a*=0.05; ***-significance level *a*=0.001; ns=no significant.

**Table S6.** ANOVA table depicting the effects of light intensity, NPK deficiency, *Amaranthus palmeri* sex and their interactions on chlorophyll fluorescence parameters

| Response variable | T | G | T×G | L×G | NPK×G | T×L×G | T×NPK×G | L×NPK×G |
| --- | --- | --- | --- | --- | --- | --- | --- | --- |
| F´_S_ | <0.0001^***^ | 0.243^ns^ | 0.266^ns^ | 0.869^ns^ | 0.0013^*^ | 0.021^*^ | 0.478^ns^ | 0.065^ns^ |
| F´_M_ | <0.0001^***^ | 0.993^ns^ | 0.924^ns^ | 0.847^ns^ | 0.0005^**^ | 0.047^*^ | 0.002^*^ | 0.079^ns^ |
| Φ_PSII_ | 0.0004^**^ | 0.227^ns^ | 0.859^ns^ | 0.837^ns^ | 0.004^*^ | 0.046^*^ | 0.021^*^ | 0.376^ns^ |

^†^T=sampling time; G=*A. palmeri* sex; L=light intensity; NPK=NPK deficiency; *=significance level *a*=0.05; ***-significance level *a*=0.001; ns=no significant.
